# Simultaneous Transepithelial Topography-Guided Photorefractive Keratectomy and Accelerated Cross-Linking in Keratoconus: 2-Year Follow-Up

**DOI:** 10.1155/2018/2945751

**Published:** 2018-10-15

**Authors:** Sibel Ahmet, Alper Ağca, Dilek Yaşa, Ahmet Alperen Koç, Mesut Toğaç, Yusuf Yıldırım, Burçin Kepez Yıldız, Ahmet Demirok

**Affiliations:** ^1^Ağrı Government Hospital, Ağrı Yolu 5. Kilometre, Doğubeyazıt, Ağrı, Turkey; ^2^Prof. Dr. N. Resat Belger Beyoglu Eye Training and Research Hospital, Bereketzade Mah. N 2, İstanbul, Turkey; ^3^Doç. Dr. Yaşar Eryılmaz Government Hospital, Fırat Mah, Erzurum Cad, Ağrı, Turkey

## Abstract

**Purpose:**

To evaluate the visual, refractive, and topographic outcomes after simultaneous topography-guided transepithelial photorefractive keratectomy (transepithelial TG-PRK) using the Amaris Excimer laser platform and accelerated corneal cross-linking (CXL) in eyes with keratoconus.

**Materials and Methods:**

Patients with 2 years of follow-up were included in this retrospective case series. Manifest refraction (MR), uncorrected (UDVA) and corrected (CDVA) distance visual acuity, corneal topography, and pachymetry were evaluated at 1, 3, 6, 12, and 24 months after surgery. The root-mean-square of total higher-order aberrations (total HOA-RMS), coma (Coma-RMS), and spherical aberration (SA-RMS) were calculated for 4- and 6-mm diameters.

**Results:**

Forty-six eyes of 46 patients were included in the study. Stromal ablation was ≤50 *μ* in all patients. MR was −3.78±3.26 preoperatively and −1.39±1.82 postoperatively. Significant improvements were seen in the UDVA and Coma-RMS values at 1 month, CDVA and total HOA-RMS values at 3 months, and SA-RMS values at 1 year compared to preoperative levels. UDVA values further improved after 2 years, compared to the 1-year values. No patient lost two or more lines and keratoconus progression was not observed in any patient.

**Conclusion:**

Simultaneous transepithelial TG-PRK and accelerated CXL resulted in significant gains in CDVA without compromising CXL efficacy.

## 1. Introduction

Keratoconus is a noninflammatory (and usually bilateral) ectatic disease of the cornea that results in localized corneal thinning and steepening. Corneal cross-linking (CXL) is the only treatment that can stop keratoconus progression [1, 2]. CXL lasts more than 60 minutes, during which riboflavin sensitization with ultraviolet-A (UVA) radiation increases the biomechanical rigidity of the anterior corneal stroma. Recently, researchers developed accelerated CXL protocols that speed up the procedure using higher-intensity radiation for a reduced period of time; these protocols can be applied in various clinical settings and effectively stabilize keratoconus [3]. However, various parameters have been used in these methods and no standard parameters for accelerated CXL have been established [3, 4].

Combining photorefractive keratectomy (PRK) with CXL or accelerated CXL offers keratoconus patients both stability and functional vision, with improvements in UDVA, CDVA, and topographic irregularity [5–8]. However, different excimer laser platforms have different ablation patterns and different algorithms for topography-guided ablation. Additionally, mechanical and laser debridement of the corneal epithelium (PRK versus transepithelial PRK) may result in different patterns of ablation and different clinical results. Therefore, clinical results with each excimer laser platform and each method of epithelial debridement should be examined separately.

Although many authors have reported the results of simultaneous TG-PRK and CXL, only a few studies reported outcomes at 2 years or beyond [9–11] and only one of them evaluated accelerated CXL with transepithelial TG-PRK [11]. In addition to these studies, Kymionis et al. [12] reported long-term results of combined transepithelial phototherapeutic keratectomy (PTK) and corneal collagen cross-linking for keratoconus (Cretan protocol). Grentzelos et al. evaluated combined transepithelial PTK and conventional PRK followed simultaneously by CXL (Cretan protocol plus) [13]. However, the accelerated cross-linking protocol that we use in combination with PRK has not been previously reported. Additionally, the Amaris platform and Optimized Refractive Keratectomy-Custom Ablation Manager (ORC-CAM) software were used in only a few studies with a limited number of patients and short follow-up periods. Camellin et al. [14] evaluated 37 eyes, but 31 of the eyes had follow-up periods that ranged from 4 months to 1 year. Müller and Lange [15] evaluated only nine eyes and the patients had follow-ups at 18 months.

The aim of this retrospective study was to evaluate the visual, refractive, and topographic outcomes, 2 years posttreatment, of keratoconic patients who were treated with a simultaneous transepithelial TG-PRK and accelerated CXL protocol in our clinic.

## 2. Materials and Methods

This retrospective study was conducted in accordance with the Declaration of Helsinki and approval was obtained from the institutional review board. The medical records of patients who underwent simultaneous transepithelial TG-PRK (Amaris 750S excimer laser platform and ORC-CAM software) and accelerated CXL (20-minute riboflavin soak, followed by 5-minute irradiation at a power of 18 mW/cm^2^) in our clinic between January 2015 and January 2016 were retrospectively evaluated. Patients who were older than 18 years of age and who underwent follow-up for at least 2 years were included in the study. The diagnosis of keratoconus was established using the Amsler-Krumeich classification (Table 1), based on astigmatism, corneal power, corneal transparency, and corneal thickness [16]. A Sirius 3D rotating Scheimpflug camera and topography system (Costruzioni Strumenti Oftalmici, Florence, Italy) were used to collect the measurements. The exclusion criteria were mechanical debridement of the cornea during PRK, CXL procedure with other parameters defined for this study, or associated ocular diseases that may affect measurements or visual acuity gains.

### 2.1. Preoperative and Postoperative Examinations

Preoperatively, all patients had a full ophthalmological examination, including UDVA and CDVA, manifest and cycloplegic refractions, slit-lamp evaluation, Goldman applanation tonometry, and fundoscopy examinations. Using the Sirius topography system, the following topographic and pachymetric parameters were recorded and evaluated for 4-mm pupil diameters, both preoperatively and postoperatively: minimum corneal thickness (MCT), maximum keratometry (*K* max), flattest keratometric reading (*K*1), steepest keratometric reading (*K*2), mean pupillary power (MPP), keratoconus vertex back (KVB, maximum posterior elevation), root-mean-square of the total higher-order aberrations (Total HOA-RMS), coma (Coma-RMS), and spherical aberration (SA-RMS).

Postoperatively, patients were examined on the first day after surgery and every alternating postoperative day to assess the status of corneal epithelial healing. If complete epithelialization was present, the therapeutic contact lens was removed. Afterward, the patients were examined 1, 3, 6, 12, and 24 months after surgery. UDVA and CDVA, manifest refraction (including the mean refractive spherical equivalent (MRSE)), corneal topography, and biomicroscopic examinations were performed at each visit. An automated phoropter (CV-5000, Topcon, Tokyo, Japan) and a back-illuminated 19” LED LCD monitor chart (CC-100 XP, Topcon, Tokyo, Japan) were used for visual acuity examinations. Visual acuities were converted to logMAR for statistical analysis. Corneal haze was recorded by Fantes haze grading system using slit lamp.

### 2.2. Surgical Technique

All PRK procedures were performed using the Schwind AMARIS 750 excimer laser platform in transepithelial PRK mode. This is a flying-spot excimer laser platform with a repetition rate of 750 Hz. The ablation profile was planned using the integrated ORC-CAM software. The treatment was planned in PRK mode to clearly see the ablation profile and stromal ablation depth. Attempted refraction was reduced to limit the ablation depth to 50 *μ* (over the cone) in the TG-PRK mode. These parameters were used for a* transepithelial* TG-PRK treatment. In the transepithelial PRK mode, the excimer laser performs epithelial and stromal ablation in one step. Mitomycin C was not used.

Transepithelial TG-PRK was followed by accelerated corneal CXL. The exposed stroma was soaked with 0.1% riboflavin (VibeX Rapid; Avedro Inc., Waltham, MA, USA) for 20 minutes, and then CXL was performed using the Avedro KXL cross-linking platform (Avedro Inc., Waltham, MA, USA) for 5 minutes at an irradiance of 18.0 mW/cm^2^ (5.4 J/cm^2^). A hypotonic riboflavin solution (Peschke Trade GmbH, Huenenberg, Switzerland) was used in corneas thinner than 400 *μ*m (1 drop every 5 seconds until the corneal thickness reaches 400 *μ*; online pachymetry of the excimer laser platform was used to monitor the corneal thickness). One additional drop of riboflavin was applied at 2.5 minutes during irradiation. The cornea was washed using a balanced salt solution at the end of the CXL process. A bandage contact lens (Cooper Vision, Scottsville, NY) was fitted at the end of the surgery and remained in place until full reepithelialization. The contact lens was removed after epithelial healing. All patients were treated postoperatively with topical moxifloxacin (0.5%; Alcon Laboratories, Inc, Fort Worth, TX, USA) four times daily until reepithelialization and with loteprednol etabonate (0.5%; Lotemax; Abdi İbrahim İlaç Sanayi ve Tic AŞ, İstanbul, Turkey) four times daily for 2 weeks with gradual tapering over an additional 1 week. All patients used preservative-free tear substitute for at least 1 month (Refresh Plus; Allergan, Inc., Irvine, TX).

### 2.3. Statistical Analysis

Statistical analysis was performed using IBM SPSS Statistics 20 software (IBM Corp, Armonk, NY, USA). The distribution of the variables was assessed using the Shapiro–Wilk test. The mean and standard deviation (SD) were used for normal distributions in descriptive statistics; median, minimum, and maximum values were used for nonnormal distributions. Visual acuity measurements were converted to logMAR for statistical analysis. The dependent-samples *t*-test was used for the variables with normal distributions and the Wilcoxon test was used for the variables with nonnormal distributions. The level of statistical significance was set at *P* < 0.05.

## 3. Results

Forty-six eyes from 46 patients were included in the study. Thirty (65%) patients were male and 16 (35%) patients were female. The mean age was 26±5 years (min: 18 years; max: 39 years). All had stage 1 or 2 keratoconus as defined based on the Amsler-Krumeich classification (20 eyes Grade 1, 26 eyes Grade 2). Demographic data and patient characteristics are shown in Table 2.

Table 3 shows the attempted correction and ablation characteristics.  Table 4 shows the visual acuity and manifest refraction values during follow-up. At the end of 24 months, on average, UDVA improved to 0.40 logMAR (max: 0.00, min: 1.00), compared with the preoperative UDVA of 0.70 logMAR (max: 0.15, min: 1.30). Additionally, on average, CDVA improved to 0.15 logMAR (max: 0.00, min: 1.00) at 24 months postsurgery compared with the presurgery value of 0.40 logMAR (max: 0.00, min: 1.00).

While there was no statistically significant difference in the mean CDVA value at 1 month, statistically significant improvements were observed at 3, 6, 12, and 24 months postsurgery, compared to preoperative values. There was also a statistically significant improvement in the MR at 1 month.

The pre- and postoperative corneal topography parameters are shown in Table 5. The changes in K1, K2, and MPP were statistically significant at all visits compared to the preoperative values.

Table 6 shows the preoperative and postoperative corneal pachymetry values. The mean preoperative pachymetry (thinnest) was 470.02 ± 31.55 *μ*m (min: 406, max: 529) and it was 414.94 ± 45.22 *μ*m (min: 311, max: 496) at 24 months after surgery (p<0.001).

The changes in the patients' higher-order aberrations during follow-up are summarized in Tables 7 and 8. Two years after surgery, statistically significant changes were found in the total HOA-RMS, Coma-RMS, and SA-RMS values at both the 4-mm and 6-mm diameters.

At 24 months after surgery, no patient had corneal melting, scarring, or perforation; HSV reactivation; or keratoconus progression. No patient experienced a clinically significant complication. Two years after surgery, there was an area of corneal opacity in 7 (15%) of the eyes. However, none of these patients had a decrease in CDVA (Table 8).

**Figure 1:**
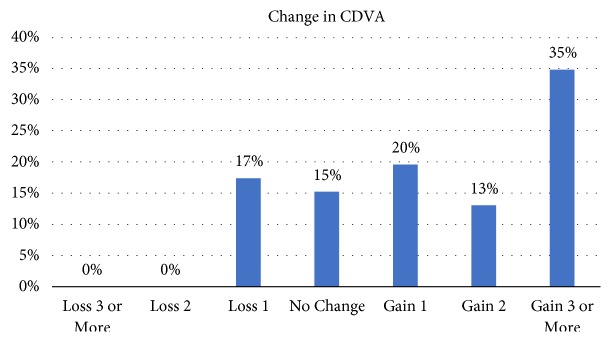
Change in corrected distance visual acuity (CDVA).

Two years after surgery, no patient lost ≥2 line of CDVA. The change in CDVA lines is shown in Figure 1.

## 4. Discussion

Although some CXL protocols result in more cross-linking and a greater increase in Young's modulus [17], less corneal cross-linking (a smaller increase in Young's modulus) may still have the same* clinical* effect (in terms of disease stabilization). In other words, the optimum CXL parameters to produce the* clinically* desired effect (in terms of stabilization or prophylaxis) are not known. For prophylactic CXL performed simultaneously with PRK, the issue becomes more complicated because defining the optimum prophylactic parameters is a greater challenge when even the optimum stabilizing parameters are not defined. Additionally, most eyes with stable keratoconus are also stable after a PRK procedure even if cross-linking is not applied, which further increases the complexity of this issue [18, 19]. Consistent with published reports, keratoconus progression was not seen in any patient during the postoperative follow-up period in our study [5–15, 18–21]. It should be noted that a non-TG PRK is associated with a decrease in HOA and an increase in CDVA [19], and we believe that non-TG PRK is a valuable option for some patients to limit the ablation depth and to use a less complicated ablation profile. With so many parameters (PRK, transepithelial-PRK, TG-PRK, and transepithelial TG-PRK in combination with different CXL protocols) and a relatively stable disease, the optimum parameters for PRK and simultaneous CXL are difficult to determine without large-scale, comparative, randomized trials with an extended duration of follow-up to establish the long-term stability of this procedure in keratoconus treatment. Although these large-scale studies have not been performed, we believe that each study with different parameters adds valuable information to get closer to identifying the optimum parameters.

In this study, we evaluated same-day simultaneous TG-transepithelial-PRK and accelerated CXL treatment in 46 patients with frequent follow-up visits for 2 years. A different accelerated CXL protocol was used with a reduced soak time, which was previously not used in combination with PRK. All patients were examined four times during the first year and all patients were examined at 2 years. This follow-up schedule is routine in our refractive surgery clinic, which is in a tertiary referral eye hospital. Every patient was examined by an ophthalmology resident first, then a specialist, and then a faculty member. Thus, although the number of patients was not high, there were no missing pieces of data during the 2-year follow-up, and early changes in visual acuity and corneal topography parameters, including high-order aberrations, during the first year are described clearly.

A reduction in HOA after TG-PRK in keratoconus eyes (with or without the addition of CXL) is not surprising and is a consistent finding in published studies. In this study, total HOA was significantly lower than its preoperative levels and total HOA at 24 months was significantly lower than it was 12 months after surgery. Coma, which is usually a significant aberration in keratoconus eyes, was reduced significantly at the 1-month visit. The mean manifest refraction was −3.78±3.26 D preoperatively and −1.39±1.82 D postoperatively; however, the results were distributed over a wide range and MRSE has, by definition, limited value in describing refractive error in a patient with irregular astigmatism. Additionally, it is difficult to perform subjective refraction in patients with irregular astigmatism because large dioptric changes may induce only a minor change in the patient's visual acuity, and therefore it is common for patients' verbal responses to be inconsistent. With these limitations in mind, MRSE values were significantly decreased after the treatment (p<0.001). As a result of the decreased MRSE and HOA values, both UDVA and CDVA improved significantly after TG-transepithelial PRK and accelerated CXL. The mean CDVA at 3 months was better than that at 1 month (p=0.01) and mean CDVA at 12 months was better than that at 6 months (p=0.003). The effect of simultaneous UDVA treatment was more striking than its effect on CDVA. The best way to assess* mean* line loss or gains in a group of patients is to evaluate logMAR visual acuity. An improvement of 1 log unit (a 0.1* increase* in the logMAR value) means a loss of one ETDRS line. It also means that the minimum angle of resolution is multiplied by 1.2589^1^. However, a 3-log-unit improvement (0.3* decrease* in logMAR) means that the minimum angle of resolution is multiplied by 1/1.2589^3^, resulting in a 50% decrease in the minimum angle of resolution (doubling of visual acuity). The mean UDVA improvement in this study was 3.5 log units (0.77 logMAR preoperatively and 0.42 logMAR postoperatively). Additionally, UCVA values at 24 months were better than UCVA values at 12 months (p=0.04). These changes in CDVA and UDVA show that corneal remodeling and visual acuity improvement continues during the first 2 years after surgery.

To the best of our knowledge, only a few transepithelial TG-guided PRK-CXL studies report outcomes at 2 years or beyond. With the exception of the study performed by Kanellopoulos et al., all of the studies had fewer patients than this study. Additionally, the CXL protocol (accelerated or not), PRK or PTK method (transepithelial or not, topography guided or not, excimer laser platform), or both differed between our study and the other studies in the literature [9–15].

Alessio et al. [9] reported outcomes after a 2-year follow-up of simultaneous transepithelial TG-PRK and CXL in 17 eyes. They performed CXL in one eye and simultaneous TG-PRK and CXL on the contralateral eye; however, they used a standard protocol for CXL (30-minute soak, 30-minute irradiation at 5.4 J/cm^2^) and they used the iVIS Suite custom ablation system and Corneal Interactive Programmed Topographic Ablation (CIPTA) planning software for transepithelial TG-PRK. Consistent with our study, they reported that an improvement in visual acuity continued beyond 1 year after the operation. Kontadakis et al. [10] reported outcomes of a 3-year follow-up after simultaneous TG-PRK and CXL in 30 eyes. Their results are consistent with our study and report an improvement of 2.7 log units in UDVA and an improvement of 0.9 log units in CDVA at 3 years. However, in contrast to our study, they used the standard CXL protocol (30-minute riboflavin-A soak, 30-minute irradiation at 5.4 J/cm^2^). A solid-state excimer laser with a wavelength of 213 nm with a Pulzar Z1 Excimer laser platform was used for a transepithelial custom ablation profile. Kymionis et al. [12] reported the outcomes of simultaneous transepithelial PTK (Allegretto Wavelight excimer laser, Wavelight Laser Technologie AG) and the standard CXL protocol in 23 eyes (16 of these eyes had follow-up periods of more than 2 years). In addition to that, Grentzelos et al. reported that combined transepithelial PTK and conventional PRK followed simultaneously by CXL (Cretan protocol plus) results in favorable outcomes in keratoconus patients [13]. Kanellopoulos et al. [11] reported the largest series with the longest follow-up using accelerated CXL and transepithelial TG-PRK. The term “accelerated” has a broad definition and different protocols of accelerated cross-linking have been used to treat keratoconus. Kanellopoulos et al. used a 30-minute riboflavin soak and an irradiation power of 10 mW/cm^2^ in contrast to our study (20-minute riboflavin soak and an irradiation power of 18 mW/cm^2^). Consistent with our study, the average gain/loss in visual acuity was consistently positive, starting from the first postoperative month, with gradual and continuous improvement toward the 3-year visit. Although we used a different CXL protocol and a different ablation pattern (Schwind Amaris ORC-CAM), our results are comparable to the studies above [9–13].

Diffuse corneal haze, which emerges in a few days after CXL in virtually all eyes, generally resolves over time. It is identified as a dust-like change in the stroma. In addition, some patients suffer from scarring that persists and can decrease visual acuity. However, these two words— “haze” and “scar”—are sometimes used interchangeably in the literature [19, 22]. No histopathological studies have evaluated these two types of opacities (haze and scar) after cross-linking and the distinction between a very mild superficial scar and a dense localized haze is not clear in the literature. In our clinical practice, we prefer to use the word “scar” to define a localized area of dense corneal opacity (an opaque patch) that is usually accompanied by a localized decrease in corneal thickness in the early postoperative period that may distort the topographical pattern. Similar to haze, the scars also fade appreciably within the first postoperative year. The effect of the opacity (haze or scar) depends on its location, severity, and the corresponding change in topography [23]. To avoid confusion, in this study we report persistent (2 years) localized corneal opacities as a complication. Two years postoperatively, seven patients (15%) in our study had still mild localized areas of central/paracentral corneal opacities. However, CDVA was increased in all of these patients (Table 9).

The most important limitation of this study is its retrospective nature and lack of a control group. In addition, slit-lamp examinations were performed by different residents at different visits. These should be considered when interpreting the results. For example, there was no objective measure of corneal haze before and after treatment because it was not a routine part of our preoperative and postoperative examinations. In addition to that, endothelial cell density (ECD) was not assessed in our patients after the operation; as a result, it was not possible to report ECD changes. Despite these limitations, this retrospective case series describes, for the first time, the clinical results of transepithelial TG-PRK using the Amaris excimer laser platform and ORC-CAM's ablation profile with a simultaneous accelerated CXL protocol in keratoconus patients, and a relatively high number of patients were included with a long follow-up.

In conclusion, we found that the transepithelial TG-PRK with the Amaris Excimer laser platform and simultaneous accelerated CXL (20-minute riboflavin soak time, 5-minute 18 mW/cm^2^ UVA irradiation) was safe and effective for treating keratoconus patients. However, large-scale, comparative, randomized trials are required to determine the optimum parameters and most suitable patients.

## Figures and Tables

**Table 1:** Amsler-Krumeich clinical classification of keratoconus by stage.

Stage	Characteristics
I	Eccentric steepening
	Induced myopia and/or astigmatism of ≤5.00 D
	Keratometric reading ≤48.00
	Vogt's lines, typical topography

II	Induced myopia and/or astigmatism 5.00 to ≤8.00 D
	Keratometric reading ≤53.00
	Pachymetry ≥ 400 *μ*m

III	Induced myopia and/or astigmatism 8.00 to ≤10.00 D
	Keratometric reading >53.00
	Pachymetry 200 to 400 *μ*m

IV	Refraction not measurable
	Keratometric reading >55.00 D
	Central scars
	Pachymetry ≤ 200 *μ*m

The patient is at the specific stage if one of the characteristics applies. Pachymetry is measured at the thinnest site of the cornea.

D, diopters.

**Table 2:** Preoperative patient characteristics.

	**Median**	**Minimum**	**Maximum**
UDVA (logMAR)	0.70	0.15	1.30
CDVA (logMAR)	0.40	0.00	1.00
SPH (D)	-1.62	-8.00	1.50
CYL (D)	-2.50	-5.00	0.00
MRSE (D)	-3.31	-12.63	0.25
	**Mean ± SD**	**Minimum**	**Maximum**

PACHYMETRY (*μ*m)	470.02 ± 31.55	406	529
K_apex_ (D)	54.90 ± 4.81	47.34	64.71
K_1_ (D)	45.50 ± 2.84	40.57	55.60
K_2_ (D)	48.72 ± 3.08	43.83	58.57
MPP (D)	45.98 ± 2.87	41.52	53.72
KVB (*μ*m)	66.72 ± 31.94	11	139
HOA-RMS (4 mm) (*μ*m)	1.04 ± 0.57	0.15	2.58
COMA-RMS (4 mm) (*μ*m)	0.91 ± 0.54	0.10	2.35
SA-RMS (4 mm) (*μ*m)	0.19 ± 0.16	0.01	0.71
HOA-RMS (6 mm) (*μ*m)	2.52 ± 1.25	0.43	5.83
COMA-RMS (6 mm) (*μ*m)	2.17 ± 1.21	0.29	5.50
SA-RMS (6 mm) (*μ*m)	0.52 ± 0.51	0.03	1.90

UDVA: uncorrected distance visual acuity; CDVA: corrected distance visual acuity; SPH: sphere; CYC: cylinder; MRSE: spherical equivalent of manifest refraction; SD: standard deviation; K_apex_: steepest keratometry; K_1_: flat keratometry; K2: steep keratometry; MPP: mean pupillary power (4.5 mm diameter ); KVB: keratoconus vertex back (posterior elevation); HOA-RMS: root-mean-square of total higher-order aberrations; Coma-RMS: root-mean-square of coma; SA-RMS: root-mean-square of spherical aberration.

**Table 3:** Attempted correction and ablation charactheristics.

	Mean ± SD	Median	Minimum/Maximum
Optical zone diameter (mm)	5.93 ± 0.58	6	5/7

Transition zone diameter (mm)	0.72 ± 0.29	0.63	0.33/1.46

Total ablation zone (mm)	6,65 ± 0,71	6,63	5.33/8.23

Maximum ablation depth*∗* (*μ*)	76± 35	85	16/138

Central ablation depth (*μ*)	48 ± 28	62	2/96

Minimum ablation depth (*μ*)	35± 28	56	0.003/61

Ablation volume (*μ*m^3^)	1745 ±1239	2001	183/4232

Attempted sphere (D)	-1.15 ± 1.51	-1.00	-4.87/1.30

Attempted cylinder (D)	-1.10 ± 0.75	-0.81	-2.99/0

*∗*: ablation profile was evaluated by the surgeon. Maximum ablation depth was limited to 50 *μ* over the cone. Paracentral ablation was not limited.

**Table 4:** Visual acuity and manifest refraction during follow-up.

	**Preoperative**	**1 month**	**3 months**	**6 months**	**12 months**	**24 months**
**UDVA (logMAR)**						
Mean±SD	0.77 ± 0.40	0.55 ± 0.31	0.49 ± 0.28	0.48 ± 0.28	0.48 ± 0.33	0.42 ± 0.29
Median	0.70	0.52	0.52	0.52	0.40	0.40
Min/Max	0.15/1.30	0.10/1.30	0.00/1.30	0.00/1.30	0.00/1.30	0.00/1.00
p value (preop)*∗*	-	**<0.001**	**<0.001**	**<0.001**	**<0.001**	**<0.001**
p value (previous)*∗∗*	-	-	**0.033**	0.475	0.687	**0.042**

**CDVA (logMAR)**						
Mean±SD	0.36 ± 0.23	0.31 ± 0.27	0.27 ± 0.24	0.26 ± 0.23	0.21 ± 0.21	0.20 ± 0.21
Median	0.40	0.22	0.22	0.22	0.15	0.15
Min/Max	0.00/1.00	0.00/1.00	0.00/1.00	0.00/1.00	0.00/1.00	0.00/1.00
p value (preop)*∗*	-	0.125	**0.013**	**0.007**	**<0.001**	**<0.001**
p value (previous)*∗∗*	-	-	**0.010**	0.100	**0.003**	0.067

**MRSE (D)**						
Mean±SD	−3.78±3.26	−1.69±1.82	−1.41±2.20	−1.42±2.00	−1.42±1.84	−1.39±1.82
Median	−3.31	−1.19	−1.06	−1.00	−1.25	−1.25
Min/Max	−12.63/0.25	−8.25/1.38	−8.25/1.38	−8.25/1.38	−5.50/1.38	−5.50/1.38
p value (preoperative)*∗*	-	**<0.001**	**<0.001**	**<0.001**	**<0.001**	**<0.001**
p value (previous)*∗∗*	-	-	0.115	0.537	0.871	0.465

SD: standard deviation; min: minimum; max: maximum; UDVA: uncorrected distance visual acuity; CDVA: corrected distance visual acuity; MRSE: spherical equivalent of manifest refraction.

*∗*: compared to preoperative visit.

*∗∗*: compared to previous visit.

**Table 5:** Corneal topography parameters during follow-up.

	**Preoperative**	**1 month**	**3 months**	**6 months**	**12 months**	**24 months**
**K**_**apex**_ ** (D)**						
Mean±SD	54.90 ± 4.81	53.30 ± 5.25	52.54 ± 5.58	51.98 ± 5.00	52.55 ± 5.30	52.42 ± 5.37
Min/Max	47.34/64.71	45.89/63.01	40.19/68.66	40.19/62.95	43.64/63.20	43.64/63.05
p (preoperative)*∗*	-	0.202	0.011	**<0.001**	**<0.001**	**<0.001**
p (previous)*∗∗*	-	-	0.71	0.243	0.512	0.124

**K**_**1**_ ** (D)**						
Mean±SD	45.50 ± 2.84	43.53 ± 3.79	43.31 ± 2.25	43.32 ± 2.25	43.81 ± 2.97	43.77 ± 2.94
Min/Max	40.57/55.60	34.00/49.49	38.88/47.07	38.88/47.07	40.19/53.25	40.07/53.25
p (preoperative)*∗*	-	0.006	**<0.001**	**<0.001**	**<0.001**	**<0.001**
p (previous)*∗∗*	-	-	0.253	0.820	0.733	0.096

**K**_**2**_ ** (D)**						
Mean±SD	48.72 ± 3.08	47.20 ± 3.55	46.79 ± 3.01	46.81 ± 3.00	47.28 ± 3.51	47.25 ± 3.48
Min/Max	43.83/58.57	42.25/55.37	40.99/51.96	40.99/51.96	42.03/58.31	41.85/58.31
p (preoperative)*∗*	-	**0.025**	**<0.001**	**<0.001**	**<0.001**	**<0.001**
p (previous)*∗∗*	-	-	0.409	0.188	0.300	0.503

**MPP (D)**						
Mean±SD	45.98 ± 2.87	44.32 ± 3.49	44.27 ± 3.55	44.24 ± 3.47	44.19 ± 2.96	44.15 ± 2.86
Min/Max	41.52/53.72	37.63/52.47	38.04/58.00	38.04/58.00	39.87/51.82	39.87/51.82
p (preoperative)*∗*	-	**0.013**	**0.018**	**0.016**	**<0.001**	**<0.001**
p (previous)*∗∗*	-	-	0.319	0.620	0.388	0.519

**KVB (** ***μ*** **m)**						
Mean±SD	66.72±31.94	62.75±27.37	74.68±34.52	73.68±34.74	78.56±32.55	78.45±32.03
Min/Max	11/139	16/110	12/183	12/183	32/151	32/151
p (preoperative)*∗*	-	0.882	**0.003**	**0.004**	**<0.001**	**<0.001**
p (previous)*∗∗*	-	-	0.112	0.387	0.670	0.823

SD: standard deviation; min: minimum; max: maximum; K_apex_: steepest keratometry; K_1_: flat keratometry; K2: steep keratometry; MPP: mean pupillary power (4.5 mm diameter); KVB: keratoconus vertex back (posterior elevation).

*∗*: compared to preoperative visit.

*∗∗*: compared to previous visit.

**Table 6:** Preoperative and postoperative pachymetry.

Corneal pachymetry	**Preoperative**	**1 Month**	**3 Months**	**6 Months**	**12 Months**	**24 Months**
Mean±SD	470±32	403±51	401±46	404±47,11	415±46	415±45

Range (Min/Max)	406/529	313/479	318/480	318/480	311/496	311/496

p*∗*	-	<0.001	<0.001	<0.001	<0.001	<0.001

p*∗∗*	-	-	0.272	0.137	0.301	0.803

SD: standard deviation; Min: minimum; Max: maximum

*∗*: compared to preoperative visit; paired samples t-test, two-tailed.

*∗∗*: compared to previous visit; paired samples t-test, two-tailed.

**Table 7:** Higher-order aberration values (4 mm) during follow-up.

	**Preoperative**	**1 month**	**3 months**	**6 months**	**12 months**	**24 months**
**HOA-RMS (** ***μ*** **m)**						
Mean±SD	1.04 ± 0.57	0.76 ± 0.58	0.61 ± 0.38	0.62 ± 0.39	0.64 ± 0.42	0.63 ± 0.41
Min/Max	0.15/2.58	0.18/2.28	0.15/1.84	0.15/1.84	0.16/1.95	0.16/1.80
p (preoperative)*∗*	-	0.153	**<0.001**	**<0.001**	**<0.001**	**<0.001**
p (previous)*∗∗*	-	-	0.141	0.763	0.982	**0.027**

**COMA-RMS (** ***μ*** **m)**						
Mean±SD	0.91 ± 0.55	0.57 ± 0.50	0.45 ± 0.35	0.47 ± 0.35	0.50 ± 0.39	0.49 ± 0.38
Min/Max	0.10/2.35	0.06/1.69	0.03/1.64	0.03/1.64	0.03/1.69	0.03/1.52
p (preoperative)*∗*	-	**0.024**	**<0.001**	**<0.001**	**<0.001**	**<0.001**
p (previous)*∗∗*	-	-	0.240	**<0.001**	0.750	0.064

**SA-RMS (** ***μ*** **m)**						
Mean±SD	0.19 ± 0.16	0.24 ± 0.29	0.11 ± 0.16	0.11 ± 0.16	0.13 ± 0.10	0.13 ± 0.10
Min/Max	0.01/0.71	0.01/1.04	−0.40/0.62	−0.40/0.62	0.00/0.44	0.02/0.45
p (preoperative)*∗*	-	0.687	0.087	0.073	**0.008**	**0.006**
p (previous)*∗∗*	-	-	0.101	0.645	0.820	0.518

SD: standard deviation; min: minimum; max: maximum; HOA-RMS: root-mean-square of total higher-order aberrations; Coma-RMS: root-mean-square of coma; SA-RMS: root-mean-square of spherical aberration.

*∗*: compared to preoperative visit.

*∗∗*: compared to previous visit.

**Table 8:** Higher-order aberration values (6 mm) during follow-up.

	**Preop**	**1 month**	**3 months**	**6 months**	**12 months**	**24 months**
**HOA-RMS (** ***μ*** **m)**						
Mean±SD	1.04 ± 0.57	0.76 ± 0.58	0.61 ± 0.38	0.62 ± 0.39	0.64 ± 0.42	0.63 ± 0.41
Min/Max	0.15/2.58	0.18/2.28	0.15/1.84	0.15/1.84	0.16/1.95	0.16/1.80
p (preoperative)*∗*	-	0.153 *∗*	**<0.001 ** **∗**	**<0.001 ** **∗**	**<0.001 ** **∗**	**<0.001 ** **∗**
p (previous)*∗∗*	-	-	0.141 *∗*	0.763 *∗*	0.982 *∗*	**0.027 ** **∗**

**COMA-RMS (** ***μ*** **m)**						
Mean±SD	0.91 ± 0.55	0.57 ± 0.50	0.45 ± 0.35	0.47 ± 0.35	0.50 ± 0.39	0.49 ± 0.38
Min/Max	0.10/2.35	0.06/1.69	0.03/1.64	0.03/1.64	0.03/1.69	0.03/1.52
p (preoperative)*∗*	-	**0.024 ** **∗**	**<0.001 ** **∗**	**<0.001 ** **∗**	**<0.001 ** **∗**	**<0.001 ** **∗**
p (previous)*∗∗*	-	-	0.240 *∗*	<0.001 *∗*	0.750 *∗*	0.064

**SA-RMS (** ***μ*** **m)**						
Mean±SD	0.19 ± 0.16	0.24 ± 0.29	0.11 ± 0.16	0.11 ± 0.16	0.13 ± 0.10	0.13 ± 0.0
Min/Max	0.01/0.71	0.01/1.04	−0.40/0.62	−0.40/0.62	0.00/0.44	0.02/0.45
p (preoperative)*∗*	-	0.687 *∗*	0.087 *∗*	0.073 *∗*	**0.008 ** **∗**	**0.006 ** **∗**
p (previous)*∗∗*	-	-	0.101 *∗*	0.645 *∗*	0.820 *∗*	0.518 *∗*

SD: standard deviation; min: minimum; max: maximum; HOA-RMS: root-mean-square of total higher-order aberrations; Coma-RMS: root-mean-square of coma; SA-RMS: root-mean-square of spherical aberration.

*∗*: compared to preoperative visit.

*∗∗*: compared to previous visit.

**Table 9:** Preoperative and postoperative (2 years) DCVA in eyes with a localized corneal opacity.

Preoperative CDVA	Postoperative CDVA
0.4	0.9
0.6	0.6
0.7	0.4
0.7	1.0
0.4	0.1
0.3	0.7
0.8	0.9

## Data Availability

The data used to support the findings of this study are available from the corresponding author upon request.
